# Epidemiology and Transmission of Respiratory Infections in Thai Army Recruits: A Prospective Cohort Study

**DOI:** 10.4269/ajtmh.18-0219

**Published:** 2018-09-04

**Authors:** Clarence C. Tam, Kathryn B. Anderson, Vittoria Offeddu, Alden Weg, Louis R. Macareo, Damon W. Ellison, Ram Rangsin, Stefan Fernandez, Robert V. Gibbons, In-Kyu Yoon, Sriluck Simasathien

**Affiliations:** 1 Saw Swee Hock School of Public Health, National University of Singapore and National University Health Systems, Singapore, Singapore;; 2 London School of Hygiene & Tropical Medicine, London, United Kingdom;; 3 University of Minnesota, Minneapolis, Minnesota;; 4 Armed Forces Research Institute of Medical Sciences, Bangkok, Thailand;; 5 Phramongkutklao College of Medicine, Bangkok, Thailand;; 6 US Army Institute of Surgical Research, San Antonio, Texas;; 7 Phramongkutklao Hospital, Bangkok, Thailand

## Abstract

Military recruits are at high risk of respiratory infections. However, limited data exist on military populations in tropical settings, where the epidemiology of respiratory infections differs substantially from temperate settings. We enrolled recruits undertaking a 10-week military training at two Royal Thai Army barracks between May 2014 and July 2015. We used a multiplex respiratory panel to analyze nose and throat swabs collected at the start and end of the training period, and from participants experiencing respiratory symptoms during follow-up. Paired sera were tested for influenza seroconversion using a hemagglutinin inhibition assay. Overall rates of upper respiratory illness and influenza-like illness were 3.1 and 2.0 episodes per 100 person-weeks, respectively. A pathogen was detected in 96% of samples. The most commonly detected microbes were *Haemophilus influenzae* type B (62.7%) or non–type B (58.2%) and rhinovirus (22.4%). At baseline, bacterial colonization was high and included *H. influenzae* type B (82.3%), *H. influenzae* non–type B (31.5%), *Klebsiella pneumoniae* (14.6%), *Staphylococcus aureus* (8.5%), and *Streptococcus pneumoniae* (8.5%). At the end of follow-up, colonization with *H. influenzae* non–type B had increased to 74.1%, and *S. pneumoniae* to 33.6%. In the serology subset, the rate of influenza infection was 3.4 per 100 person-months; 58% of influenza infections resulted in clinical disease. Our study provides key data on the epidemiology and transmission of respiratory pathogens in tropical settings. Our results emphasize the need for improved infection prevention and control in military environments, given the high burden of illness and potential for intense transmission of respiratory pathogens.

## INTRODUCTION

Military recruits are at high risk of respiratory infections.^1^ The congregation of individuals from diverse geographic locations in semi-closed settings, together with high levels of close contact, provide conditions that favor the introduction and transmission of respiratory pathogens.^2^ Studies among military recruits have found high rates of illness and infection with respiratory viruses. A longitudinal study in Singapore found that 39.4% of military personnel had evidence of seroconversion against influenza A/H1N1 during the 2009 pandemic, compared with 13.5% of individuals in the community and 6.5% of hospital staff.^3^ Numerous outbreaks of respiratory infections among military recruits have been reported in the literature, including outbreaks of influenza, adenovirus,^4–6^ and pertussis.^7^ Resumption of vaccination against adenovirus types 4 and 7 in the U.S. military is estimated to have resulted in a 7-fold decrease in acute respiratory disease^8^ and a shift in the predominant adenovirus types from types 4, 3, and 7 to types 1 and 2.

The increasing availability of multiplex molecular diagnostic assays can yield data on multiple pathogens that would not routinely be detected.^9,10^ Studies in military populations can provide valuable information regarding the epidemiology and transmission of respiratory infections in adults because of the availability of well-defined populations that can be followed up over time. Despite this, data from tropical settings, in which the epidemiology of influenza and other respiratory infections differs substantially from temperate settings, are limited. Increased understanding of the etiology of respiratory infections in these settings is important to identify opportunities for disease control in high-risk military populations and to identify risks of pathogen emergence with potential for spread into the wider community.

We present the results of a longitudinal study of the burden and etiology of respiratory infections in Thai military recruits.

## MATERIALS AND METHODS

The study setting and procedures have been previously described.^11^ Between May 2014 and May 2015, we enrolled participants from consecutive cohorts of recruits undertaking basic military training at two Royal Thai Army (RTA) barracks in Bangkok. Trainees entered the camps at the start of May and November each year and remained in the camps for 10 weeks. The camps consist of large, common sleeping quarters with beds arranged foot-to-foot in long rows. Trainees share meals in a common canteen and train in a large field in the center of the barracks and a number of ancillary buildings. Each camp has its own medical unit.

Individuals were eligible for enrollment if they were aged ≥ 18 years and were entering one of the two army barracks involved in the study. Suspected tuberculosis cases or individuals with immune deficiencies, such as acquired immune deficiency syndrome, leukemia, or lymphoma, were excluded. Individuals providing informed consent were enrolled in the study and a demographic and clinical questionnaire was administered. Participants were then followed up for symptoms of respiratory illness until the end of the 10-week training period. At one camp, nasal and throat swabs were obtained at the time of enrolment and at the end of the follow-up period (10 weeks later) from consenting participants. Participants experiencing respiratory symptoms were asked to consult the medical unit, where medical staff took a history, conducted a medical examination, and recorded symptoms of upper respiratory illness (URI) or influenza-like illness (ILI). Upper respiratory illness was defined as an illness with at least two of the following: 1) runny nose or sneezing; 2) nasal congestion; 3) sore throat, hoarseness, or difficulty swallowing; 4) cough; 5) swollen or tender glands in the neck; and 6) fever (oral temperature > 38°C) at the time of presentation. Influenza-like illness was defined as a respiratory illness with acute onset presenting with fever and cough or sore throat. All ILI cases also met the case definition for URI. Non-ILI cases were defined as URI cases not meeting the case definition for ILI. Additional nasal and throat swabs were requested from participants presenting with acute ILI or URI.

### Laboratory investigations.

Nasal and throat swabs were placed in viral transport media and stored at −20°C until transfer to the Armed Forces Research Institute of Medical Sciences for further testing. We tested acute swabs for influenza virus using reverse transcription polymerase chain reaction (RT-PCR) following the U.S. Centers for Disease Control protocol.^12,13^ We also tested acute samples (from both camps) and the routine enrolment and follow-up specimens (from one camp) using a multiplex real-time PCR assay comprising 33 bacterial, viral, and fungal targets (FTD33 kit; Fast Track Diagnostics, Esch-sur-Alzette, Luxembourg). A cycle threshold value of < 33 was considered a positive result. Previous studies have shown high agreement between FTD33 and other commercial multiplex assays with specificities > 95% for most viral targets.^14,15^ Sensitivity for influenza A and B, and human coronaviruses is high (> 90%), but is lower and more variable for other viral targets, including human bocavirus (80–92%), rhinovirus (61–75%), respiratory syncytial virus (50–72%), and human metapneumovirus (74–100%).^14,15^

In a subset of recruits, we measured influenza antibody titers in paired sera taken at enrollment and at the end of follow-up using a hemagglutinin inhibition assay. Seroconversion was defined as a 4-fold rise in influenza subtype-specific antibody titers.

### Statistical analysis.

#### Rates of illness.

Individuals were considered at risk from the date they entered the camp for a period of 10 weeks. We computed the rates of URI and ILI as the number of cases of each outcome divided by the total person-time at risk, using robust standard errors in the calculation of 95% confidence intervals (CIs) to allow for clustering of illness episodes by camp and cohort.

#### Disease etiology.

We determined the percentage of URI and ILI cases positive for each target pathogen, the percentage positive for more than one pathogen, and the percentage with no pathogen identified.

#### Influenza seroconversion.

We calculated the percentage of recruits who seroconverted, overall and by influenza subtype, based on a 4-fold rise in antibody titer between baseline and end of follow-up samples.

#### Bacterial colonization.

We determined the percentage of recruits with a positive identification of a bacterial pathogen at recruitment, the overall change in colonization prevalence between baseline and end of follow-up for each bacterial species, and the risk of acquisition of bacterial pathogens over the follow-up period among those initially negative.

#### Antibiotic use.

We calculated the percentage of URI and ILI cases that were prescribed antibiotics and investigated clinical signs and symptoms associated with antibiotic prescription using logistic regression. Among those prescribed antibiotics, we determined the fraction of cases with a likely viral etiology based on the available Fast Track multiplex PCR results.

Analyses were conducted using Stata 11 (StataCorp, College Station, TX) and R 3.2.2.

### Ethical approval.

The study was approved by the Institutional Review Boards of the RTA in Bangkok, Thailand, the Walter Reed Army Institute of Research, and the London School of Hygiene & Tropical Medicine. All participants provided written informed consent.

## RESULTS

Three cohorts of army recruits undertook basic military training at the two camps during the study period, in May 2014–July 2014, November 2014–January 2015, and May 2015–July 2015. In total, 933 recruits undertook training during these three periods. Of these, 867 (93%) were enrolled in the study. Of the 867 participants, five participants withdrew from the study and 44 participants recruited in November 2014 were transferred out of the camp to complete their training elsewhere. The remaining 818 (94%) completed the 10-week follow-up and were included in the analysis. All participants were male and the median age was 21 years (range: 20–29 years). Details of recruitment and follow-up completion for each cohort are given in Supplemental Figure 1.

### Rates of illness.

**Figure 1.**
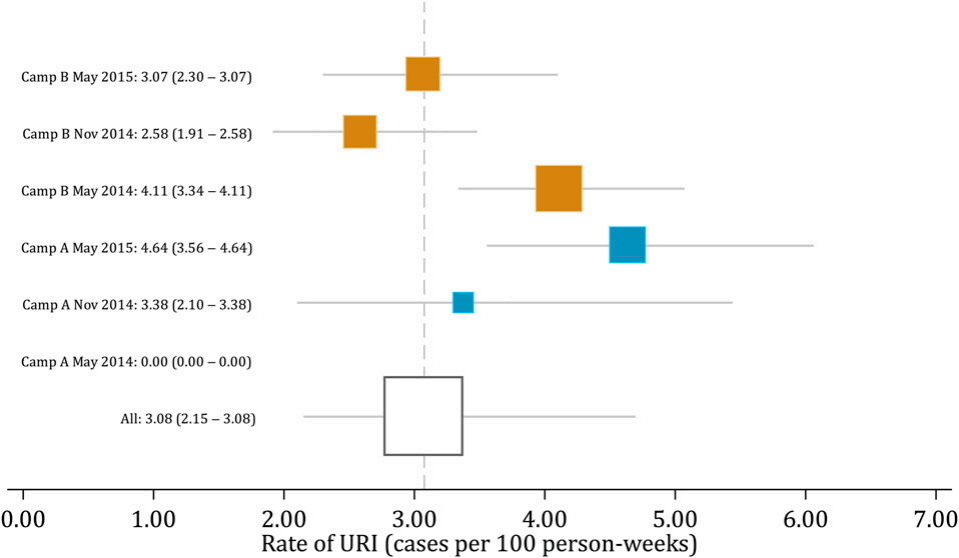
Rates of upper respiratory illness (URI) by cohort in camp A (blue boxes) and camp B (orange boxes). The area of boxes is proportional to the size of each cohort. Gray lines indicate 95% confidence intervals. No URI cases were detected in camp A during the May 2014 training period. This figure appears in color at www.ajtmh.org.

There were a total of 248 episodes of URI and 8,063 person-weeks of follow-up, yielding an overall URI rate of 3.1 per 100 person-weeks. Upper respiratory illness rates ranged from 2.6 to 4.6 per 100 person-weeks across cohorts, with the exception of the camp A May 2014 cohort, in which no URI episodes were reported (Figure 1). There were 160 ILI episodes, giving an overall ILI rate of 2.0 per 100 person-weeks, ranging from 0 to 4.1 per 100 person-weeks across cohorts.

### Symptoms and time off work.

Fever was present in nearly two-thirds of URI episodes. Cough and sore throat occurred in > 90% of URI episodes, nasal congestion and headache in > 80% of episodes, and malaise and breathing difficulty in > 60% of episodes. All these symptoms were significantly more common in ILI compared with non-ILI episodes (Table 1). Influenza-like illness cases were also more likely to take time off work than non-ILI cases (57.5% versus 13.6%, *P* < 0.001), although the length of time taken off work was short (median 1 day). There was one hospitalization in an individual diagnosed with pneumonia who was off work for 7 days.

**Table 1:** Distribution of symptoms in URI and ILI cases among Royal Thai Army recruits

Symptom	URI (*N* = 248)		ILI (*N* = 160)		Non-ILI (*N* = 88)		*P* value*
	Number	(%)	Number	(%)	Number	(%)	
Fever	160	64.5	160	100.0	0	0.0	< 0.001
Cough	236	95.2	157	98.1	79	89.8	0.005
Sore throat	232	93.5	156	97.5	76	86.4	0.002
Nasal congestion	204	82.3	154	96.3	50	56.8	< 0.001
Headache	209	84.3	146	91.3	63	71.6	< 0.001
Malaise	171	69.0	143	89.4	28	31.8	< 0.001
Breathing difficulty	156	62.9	139	86.9	17	19.3	< 0.001
Muscle ache	141	56.9	136	85.0	5	5.7	< 0.001
Tonsillitis	55	22.2	50	31.3	5	5.7	< 0.001
Swollen lymph nodes	50	20.1	43	26.9	7	8.0	< 0.001
Paralysis	42	16.9	40	25.0	2	2.3	< 0.001
Chills	37	14.9	36	22.5	1	1.1	< 0.001
Diarrhea	16	6.5	14	8.8	2	2.3	0.058
Stiffness	1	0.4	1	0.6	0	0.0	1.000
Vision or hearing loss	1	0.4	1	0.6	0	0.0	1.000
Impact on daily duties:							
Stopped working	104	41.9	92	57.5	12	13.6	< 0.001
Assigned lighter duties	71	28.6	38	23.8	33	37.5	–
Days off work (median [IQR†])	0 (0–1)	–	1 (0–1)	–	–	–	< 0.001†

ILI = influenza-like illness; URI = upper respiratory illness.

* Fisher’s exact test comparing ILI and non-ILI cases.

† IQR = interquartile range.

### Disease etiology.

Among the 134 URI and ILI samples tested by multiplex PCR, a pathogen was detected in 128. A single pathogen was detected in 26% of samples, two pathogens in 31%, three pathogens in 26%, and four pathogens in 12%. Among URI pathogens, the most common viruses detected were rhinoviruses (22.4%), influenza B (11.9%), coronavirus 229 (5.2%), adenovirus (3.0%), and influenza A/H3 (3.0%). Other viruses accounted for < 3% of URI cases each. Of 133 samples tested for influenza by both RT-PCR and the Fast Track multiplex assay, 20 (15%) tested positive by at least one assay, and all of these were also positive by RT-PCR. Of the 20 RT-PCR positives, four were positive for influenza A/H3, of which one was also positive by the multiplex assay. Of the remaining 16 positive for influenza B by RT-PCR, 14 were also positive by the multiplex assay. There were no samples that tested positive by the multiplex assay and negative by RT-PCR.

Among the bacteria, the most commonly detected species were *Haemophilus influenzae* type B (62.7%), *H. influenzae* non–type B (58.2%), *Streptococcus pneumoniae* (16.4%), and *Klebsiella pneumoniae* (16.4%). However, these were commonly found in combination with other pathogens. When considering only samples in which a single organism was found, *H. influenzae* type B was found in 13.4% of samples, *H. influenzae* non–type B in 9.0%, and *S. pneumoniae*, *K. pneumoniae*, and *Legionella pneumophila* in 0.7% each. There was little evidence to suggest that etiological agents differed between ILI and non-ILI cases, with the exception of *H. influenzae* non–type B and coronavirus 229, which occurred more frequently in non-ILI cases, and influenza A/H3 and coronavirus HKU1, which occurred more frequently in ILI cases (Table 2).

**Table 2:** Distribution of respiratory pathogens in a subset of URI and ILI cases among Royal Thai Army recruits tested by Fast Track multiplex respiratory panel (FTD33)

Pathogen type		URI (*N* = 134)		ILI (*N* = 48)		Non-ILI (*N* = 86)		*P* value*
	Pathogen	Positive	%	Positive	%	Positive	%	
Bacteria	*Haemophilus influenzae B*	84	62.7	35	72.9	49	57.0	0.093
	*H. influenzae non-B*	78	58.2	16	33.3	62	72.1	< 0.001
	*Klebsiella pneumoniae*	22	16.4	11	22.9	11	12.8	0.149
	*Streptococcus pneumoniae*	22	16.4	5	10.4	17	19.8	0.225
	*Moraxella catarrhalis*	8	6.0	3	6.3	5	5.8	1.000
	*Staphylococcus aureus*	7	5.2	2	4.2	5	5.8	1.000
	*Legionella pneumophila*	4	3.0	2	4.2	2	2.3	0.617
Viruses	Rhinovirus	30	22.4	7	14.6	23	26.7	0.132
	Influenza B (PCR/FT)†	16	11.9	5	10.4	11	12.8	0.786
	Coronavirus 229	7	5.2	0	0.0	7	8.1	0.050
	Adenovirus	4	3.0	0	0.0	4	4.7	0.296
	Influenza A H3 (PCR/FT)†	4	3.0	4	8.3	0	0.0	0.015
	Coronavirus HKU1	3	2.2	3	6.3	0	0.0	0.044
	Coronavirus 63	2	1.5	0	0.0	2	2.3	0.537
	Parainfluenza 2	2	1.5	0	0.0	2	2.3	0.537
	Parainfluenza 4	2	1.5	0	0.0	2	2.3	0.537
	Coronavirus 43	1	0.7	1	2.1	0	0.0	0.358
Negative	–	6	4.5	5	10.4	1	1.2	0.022

ILI = influenza-like illness; PCR = polymerase chain reaction; URI = upper respiratory illness.

* Fisher’s exact test comparing ILI and non-ILI cases.

† Tests positive by either reverse transcription polymerase chain reaction or Fast Track multiplex PCR.

**Figure 2.**
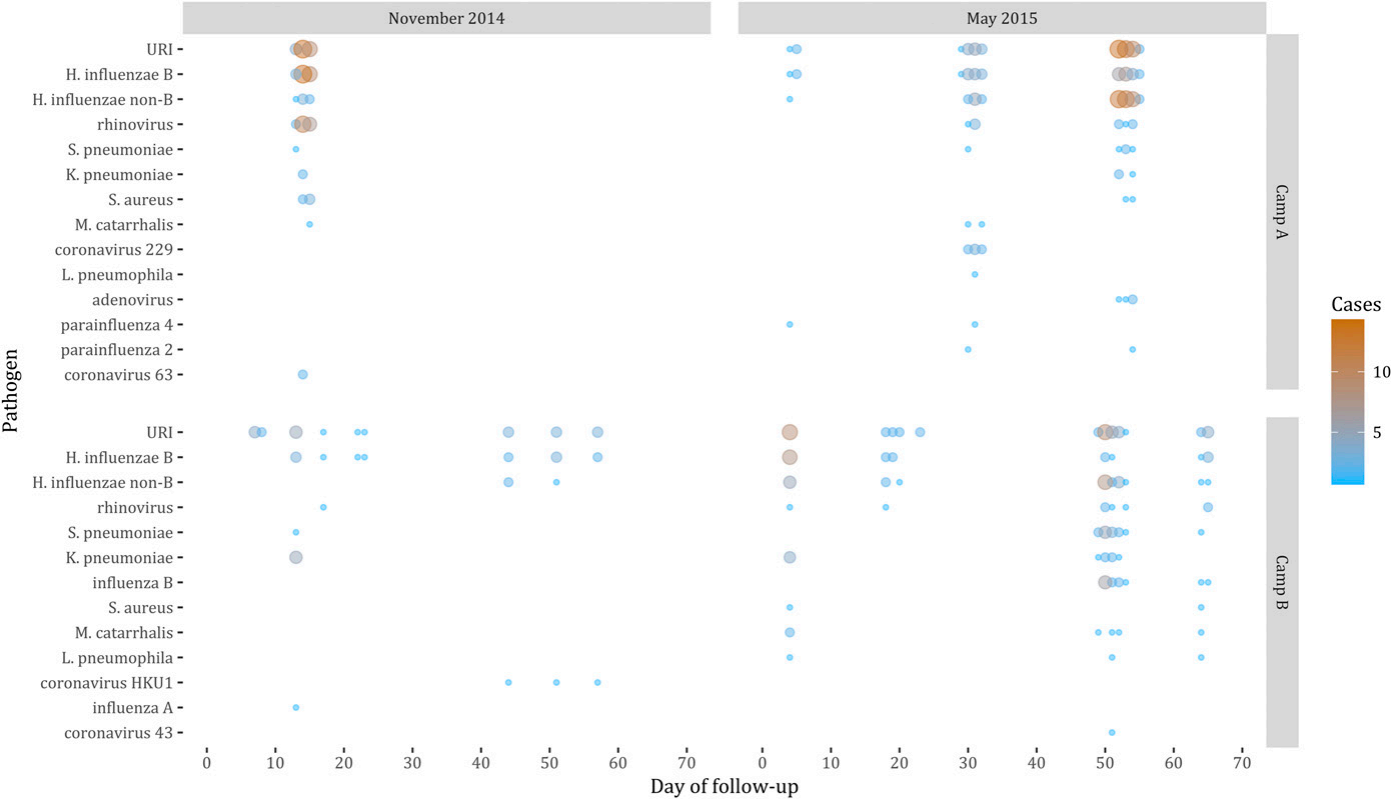
Pathogens detected in upper respiratory illness (URI) cases by camp, training cohort, and day of onset relative to the start of follow-up. Size and color of circles indicate number of URI cases in which a given pathogen was detected. This figure appears in color at www.ajtmh.org.

Analysis of URI cases by time since the start of follow-up indicated strong temporal clustering of cases, with each cluster involving several respiratory pathogens (Figure 2). For example, the single cluster in Camp A during the November 2014 cohort resulted in identification of both rhinovirus and coronavirus 63, as well as numerous colonizing bacteria. In other clusters, rhinovirus was also identified in combination with parainfluenza viruses, adenovirus, and influenza B virus (Figure 2).

### Influenza vaccination and seroconversion.

**Figure 3.**
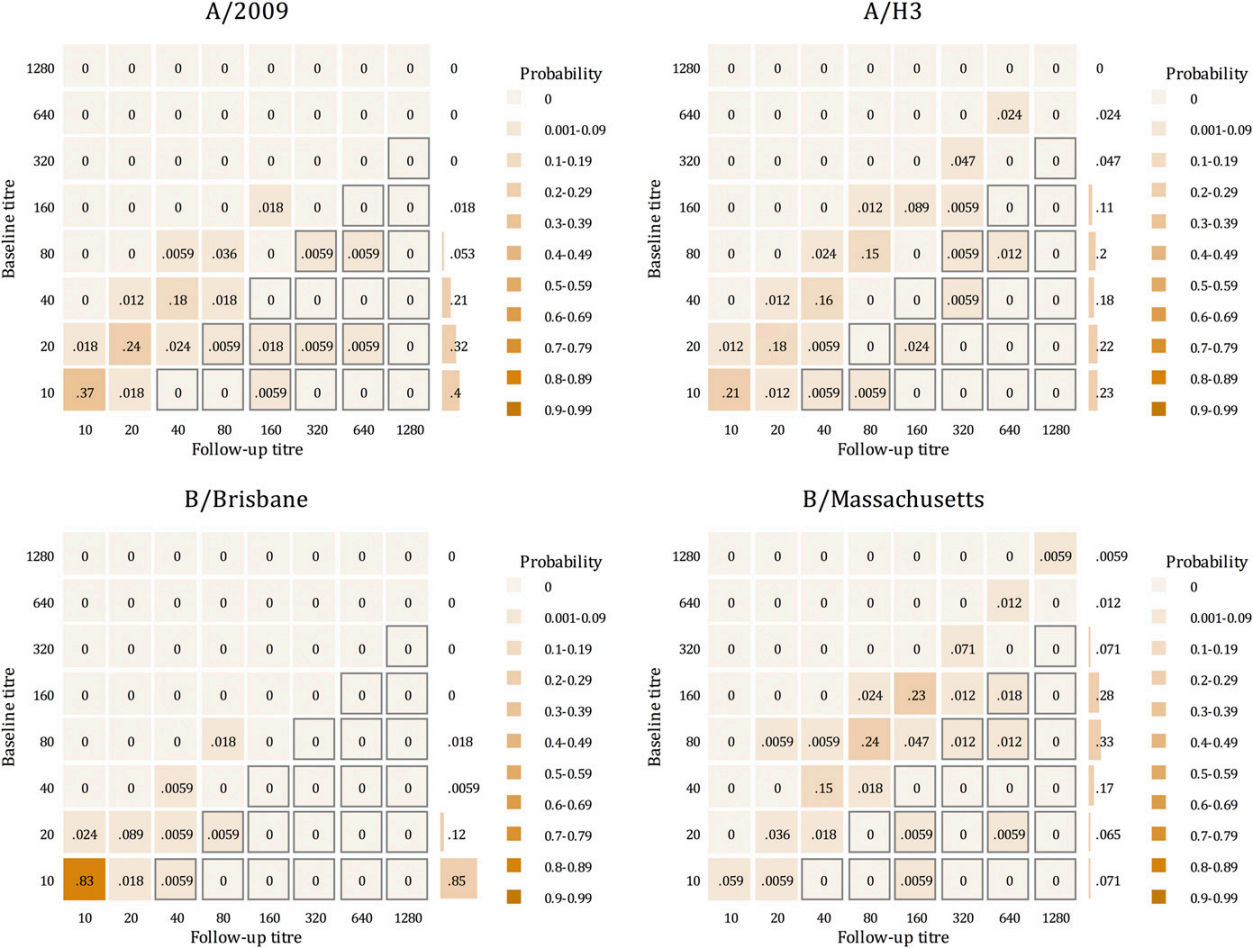
Distribution of antibody titers as measured by hemagglutinin inhibition assay at baseline (*y* axis) and follow-up (*x* axis). The color scale indicates the proportion of sample pairs in each titer combination. Gray squares denote a ≥ 4-fold increase in antibody titer at follow-up. Row totals indicate the probability distribution of baseline titers. This figure appears in color at www.ajtmh.org.

At enrollment, 2.4% of recruits reported having received influenza vaccination in the previous 12 months. In the serology subset, the rate of influenza infection was 3.4 per 100 person-months (Table 3). Thirteen of 169 (8%) recruits seroconverted over the 10-week training period. Seroconversion was most common against influenza B/Massachusetts (*N* = 7) and influenza A/H3 (*N* = 3) (Table 3). Median baseline titers were 1:80 (IQR: 1:40–1:160) for influenza B/Massachusetts, 1:40 (IQR: 1:20–1:80) for influenza A/H3, 1:20 (IQR: 1:10–1:40) for influenza A/pdmH1N1, and 1:10 (IQR: 1:10–1:10) for influenza B/Brisbane. There was a strong correlation between baseline and end of follow-up antibody titers, with evidence of low prior exposure to B/Brisbane but high prior exposure to B/Massachusetts (Figure 3). Comparison of overall seroconversion and clinical influenza rates indicated that approximately 58% of influenza infections resulted in clinical disease.

**Table 3:** Influenza seroconversion rates per 100 person-months among two cohorts of Royal Thai Army recruits entering basic training

Influenza strain	Cohort 1* (*N* = 51)		Cohort 2† (*N* = 118)		Total (*N* = 169)	
	*N*	Rate (95% CI)	*n*	Rate (95% CI)	*n*	Rate (95% CI)
*A/2009*	0	–	2	0.75 (0.19; 3.00)	2	0.52 (0.13; 2.09)
*A/H3*	1	0.87 (0.12; 6.15)	2	0.75 (0.19; 3.00)	3	0.78 (0.25; 2.43)
*B/Massachusetts*	0	–	7	2.62 (1.25; 5.50)	7	1.83 (0.87; 3.84)
A (unclassified)	0	–	1	0.37 (0.05; 2.66)	1	0.26 (0.04; 1.86)
Any	1	0.87 (0.12; 6.15)	12	4.50 (2.55; 7.92)	13	3.40 (1.97; 5.86)

CI = confidence interval.

* November 1, 2014–January 9, 2015.

† May 1, 2015–July 9, 2015.

### Colonization.

At baseline, 82.3% of recruits were colonized with *H. influenzae* type B and 31.5% with *H. influenzae* non–type B. Other common colonizing bacteria were *K. pneumoniae* (14.6%), *Staphylococcus aureus* (8.5%), and *S. pneumoniae* (8.5%). At the end of follow-up, colonization with *H. influenzae* non–type B had increased to 74.1%, and *S. pneumoniae* to 33.6%, whereas colonization with *H. influenzae* type B and *K. pneumoniae* had decreased to 67.2% and 8.4%, respectively. In addition, *Moraxella catarrhalis* was found in four (3.1%) and *L. pneumophila* in two (1.5%) end of follow-up samples. No change in *S. aureus* colonization was found. In a subset of paired baseline and end of follow-up samples initially negative for *H. influenzae* non–type B (*N* = 89), the risk of *H. influenzae* non–type B acquisition was 70.1% (95% CI: 54.4–90.6%). Among those negative for *S. pneumoniae* at baseline (*N* = 119), the risk of acquiring *S. pneumoniae* during the follow-up period was 31.9% (95% CI: 22.6–43.8%).

### Use of medications.

Of 248 URI episodes, 39.1% were prescribed antibiotics. Of these, all but one were prescribed amoxicillin or azithromycin. In multivariable logistic regression, cases were more likely to be prescribed antibiotics if they presented with tonsillitis (OR = 5.10, 95% CI: 2.35–11.10), chills (OR = 3.91, 95% CI: 1.44–10.62), and swollen lymph nodes (OR = 2.54, 95% CI: 1.07–6.04). Antibiotic prescription was less likely if patients presented with nasal congestion (OR = 0.26, 95% CI: 0.12–0.54). There was no difference in prescribing between ILI and non-ILI patients. Of 35 URI cases prescribed antibiotics and with available multiplex PCR results, 16 (45.6%) had viral pathogens detected and eight (22.9%) had a bacterial target detected in the absence of other pathogens.

## DISCUSSION

We found a high incidence of URI in Thai army recruits, indicating that approximately 30% of recruits experienced a URI episode over the 10-week training period. Comparison with other military cohorts is not straightforward because of differences between settings in terms of vaccination policies and seasonality of key pathogens such as influenza. The URI and ILI rates in our study are nonetheless comparable with those reported by other studies in the United States.^16–18^ Despite this, we found low rates of clinical influenza A in this minimally vaccinated population. Possible reasons for this could be exposure to circulating influenza strains before recruits entering the training camps. Thai national influenza surveillance data indicate high influenza activity in the first half of 2014, with influenza A/H1N1 and influenza B predominating.^19^ Concomitantly, our data indicated that 55% of recruits with available serological information had baseline influenza A/H1N1 titers ≥ 1:40. Titers greater than this value have been shown to be associated with a 50% reduction in risk of clinical influenza from homologous strains, for both influenza A and B viruses.^20–23^ Of interest, 86% of recruits also had baseline titers against influenza B/Massachusetts greater than this level. The higher baseline titers against influenza B/Massachusetts are consistent with the frequent detection of influenza B isolates from the Yamagata lineage in clinical specimens from Thailand in the first half of 2014.^19^

In our study, the Fast Track multiplex assay appeared to have lower sensitivity for influenza compared with the CDC RT-PCR assay. We did not specifically investigate reasons for these discrepancies, although it should be noted that the two assays were not performed at the same time, as the multiplex assays were all performed at the end of data collection. We therefore cannot discount the possibility that sample degradation might have influenced the relative performance of the Fast Track assay.

Although influenza incidence was low, pathogen detection with sensitive multiplex diagnostics indicated high frequencies of respiratory pathogens, with co-circulation of multiple viruses including rhinovirus, adenovirus, and coronaviruses as well as influenza. In addition, we found high levels of colonization with numerous bacterial species, including both type B and non–type B *H. influenzae*, *K. pneumoniae*, and *S. pneumoniae*. Although we did not specifically study transmission chains or epidemiologic links between cases of illness, in the context of our study, recruits did not leave the camps during the 10-week training periods, such that the occurrence of respiratory pathogens is most likely to have resulted from transmission within the camps rather than from external sources. Intense transmission of bacterial species was apparent, as the risks of acquiring *H. influenzae* non–type B and *S. pneumoniae* among initially non-colonized individuals were 70% and 31%, respectively. Bacterial species were also commonly found in samples from URI cases. The colonizing nature of these bacterial species makes it difficult to determine the clinical relevance of these detections. However, *H. influenzae* type B and non–type B were identified in 13% and 9% of clinical samples in the absence of any other pathogens, suggesting that these pathogens could be responsible for illness in a fraction of adult URI cases. Interestingly, *H. influenzae* type B has not been previously associated with a significant burden of URI in adults.^24^ It is possible, however, that these cases were caused by other pathogens not included in the multiplex assay.

High levels of colonization with bacterial species also point to the need for judicious use of antibiotics to treat URI in military populations. A quarter of URI cases in our study were prescribed broad-spectrum antibiotics, of whom 50% were more likely to have infections caused by viral pathogens based on multiplex PCR results. Although we lacked additional information to determine whether this resulted in adverse antibiotic resistance patterns, high levels of antibiotic use in a population with a high background of bacterial colonization presents a risk for development of antibiotic resistance, and highlights the importance of improved rapid diagnostics to rule out viral etiologies to aid clinical management. In addition, it points to the need for increased antibiotic stewardship and health-care provider education to reduce unnecessary use of antibiotics in military settings.

Our study also emphasizes the importance of systematic microbiological surveillance of URI in military populations, to allow prompt detection and control of outbreaks and early identification of emerging pathogens that may be circulating or could be introduced in the wider population. This is particularly important as infection control options in such closed settings are limited and vaccination coverage for key pathogens is low.

Limited resources meant it was not possible to conduct multiplex diagnostics for all baseline, acute, and end of follow-up samples, as well as influenza serology. As a result, there was limited power to investigate whether colonization influenced risk of illness, or factors associated with colonization and acquisition of bacteria. In addition, co-circulation of multiple pathogens makes it difficult to establish chains of transmission, which would require more detailed microbial sequencing information. Clinical signs and symptoms were available at presentation only, and we did not have information regarding the duration of individual symptoms or additional clinical investigations conducted longitudinally during the course of illness, as these were not routinely performed.

The specific barracks setting and population characteristics limit the generalizability of our findings to other military settings with similar conditions. Nevertheless, our study provides key data regarding the epidemiology and transmission of respiratory pathogens in military populations in tropical settings. Our results emphasize the need for improved infection prevention and control strategies in military settings, given the potential for intense transmission of a wide range of respiratory pathogens.

## Supplementary Material

Supplemental figure
