# Supplementary material for: Epidemiology and Transmission of Respiratory Infections in Thai Army Recruits: A Prospective Cohort Study
Source: Am J Trop Med Hyg. 2018 Sep 4;99(4):1089–95. doi: 10.4269/ajtmh.18-0219 (PMC6159564; doi:10.4269/ajtmh.18-0219)
Supplement: Supplementary file 1 [file tpmd180219.SD1.pdf]

The following are supplemental materials and will be published online only

Supplementary Figure 1. Number of participants and samples by camp and cohort. *Includes 41 baseline and follow-up samples from participants who had URI symptoms at the time of sample collection. **Includes 32 baseline and follow-up samples from participants who had URI symptoms at the time of sample collection.

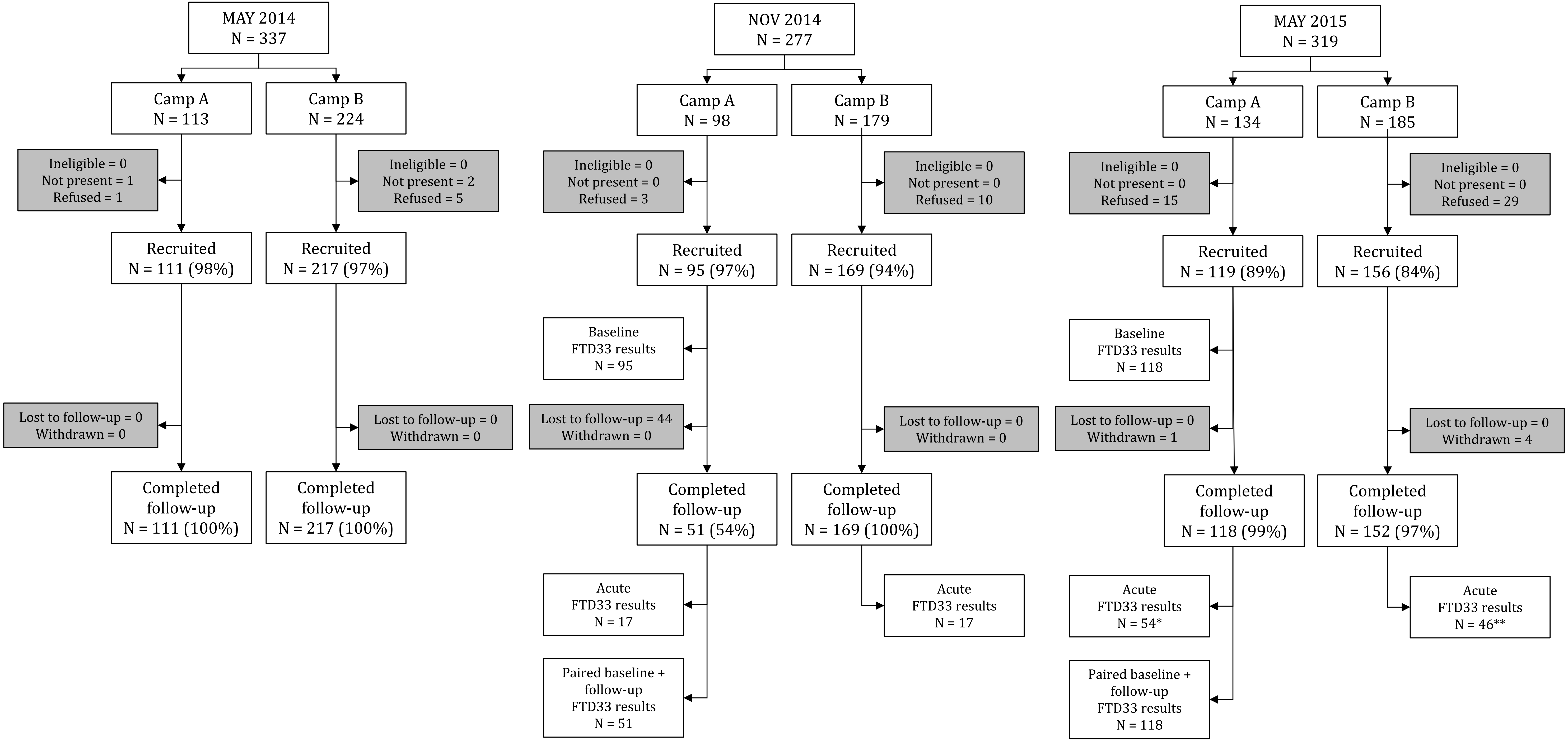
MAY 2014
N = 337
Camp A
N = 113
Camp B
N = 224
Ineligible = 0
Not present = 1
Refused = 1
Ineligible = 0
Not present = 2
Refused = 5
Recruited
N = 111 (98%)
Recruited
N = 217 (97%)
Lost to follow-up = 0
Withdrawn = 0
Lost to follow-up = 0
Withdrawn = 0
Completed follow-up
N = 111 (100%)
Completed follow-up
N = 217 (100%)
NOV 2014
N = 277
Camp A
N = 98
Camp B
N = 179
Ineligible = 0
Not present = 0
Refused = 3
Ineligible = 0
Not present = 0
Refused = 10
Recruited
N = 95 (97%)
Recruited
N = 169 (94%)
Baseline FTD33 results
N = 95
Lost to follow-up = 44
Withdrawn = 0
Lost to follow-up = 0
Withdrawn = 0
Completed follow-up
N = 51 (54%)
Completed follow-up
N = 169 (100%)
Acute FTD33 results
N = 17
Acute FTD33 results
N = 17
Paired baseline + follow-up FTD33 results
N = 51
MAY 2015
N = 319
Camp A
N = 134
Camp B
N = 185
Ineligible = 0
Not present = 0
Refused = 15
Ineligible = 0
Not present = 0
Refused = 29
Recruited
N = 119 (89%)
Recruited
N = 156 (84%)
Baseline FTD33 results
N = 118
Lost to follow-up = 0
Withdrawn = 1
Lost to follow-up = 0
Withdrawn = 4
Completed follow-up
N = 118 (99%)
Completed follow-up
N = 152 (97%)
Acute FTD33 results
N = 54*
Acute FTD33 results
N = 46**
Paired baseline + follow-up FTD33 results
N = 118
